# Metabolomics Analysis and Antioxidant Potential of Endophytic *Diaporthe fraxini* ED2 Grown in Different Culture Media

**DOI:** 10.3390/jof8050519

**Published:** 2022-05-18

**Authors:** Wen-Nee Tan, Kashvintha Nagarajan, Vuanghao Lim, Juzaili Azizi, Kooi-Yeong Khaw, Woei-Yenn Tong, Chean-Ring Leong, Nelson Jeng-Yeou Chear

**Affiliations:** 1 Chemistry Section, School of Distance Education, Universiti Sains Malaysia, Minden 11800, Penang, Malaysia; kashvintha18@gmail.com; 2 Advanced Medical and Dental Institute, Universiti Sains Malaysia, Kepala Batas 13200, Penang, Malaysia; vlim@usm.my; 3 Centre for Drug Research, Universiti Sains Malaysia, Minden 11800, Penang, Malaysia; nelsonchear@usm.my; 4 School of Pharmacy, Monash University Malaysia, Subang Jaya 47500, Selangor, Malaysia; khaw.kooiyeong@monash.edu; 5 Branch Campus Institute of Medical Science Technology (MESTECH), Universiti Kuala Lumpur, Kajang 43000, Selangor, Malaysia; wytong@unikl.edu.my; 6 Malaysian Institute of Chemical and Bioengineering Technology (MICET), Universiti Kuala Lumpur, Alor Gajah 78000, Melaka, Malaysia; crleong@unikl.edu.my

**Keywords:** antioxidant, *Diaporthe fraxini*, endophytic, LC-HRMS, metabolomics

## Abstract

Endophytic fungi are a promising source of bioactive metabolites with a wide range of pharmacological activities. In the present study, MS-based metabolomics was conducted to study the metabolomes variations of endophytic *Diaporthe fraxini* ED2 grown in different culture media. Total phenolic content (TPC), total flavonoid content (TFC), 2,2-diphenyl-1-picrylhydrazyl (DPPH) radical scavenging, 2,2-azinobis(3-ethylbenzothiazoline-6-sulfonic acid (ABTS), and ferric reducing antioxidant power (FRAP) assays were conducted to assess the antioxidant potential of the fungal extracts. Multivariate data analysis (MVDA) was employed in data analysis and interpretation to elucidate the complex metabolite profile. The supplemented culture medium of *D. fraxini* fungal extract stimulated the production of metabolites not occurring in the normal culture medium. Antioxidant activity studies revealed the potential of supplemented cultured fungal extract of *D. fraxini* as a source of antioxidants. The present findings highlight that fungal culture medium supplementation is an effective approach to unravelling the hidden metabolome in plant-associated fungal diversity.

## 1. Introduction

The plant kingdom houses a diverse group of beneficial endophytic species. Endophytes inhabit host plant tissues without causing any apparent disease symptoms to them. This plant–endophyte relationship is mutualistic and important in plant micro-ecosystems. The host provides habitation and nutrients for endophytes to complete their life cycles. Meanwhile, endophytes enhance the host’s ability to tolerate biotic and abiotic stress conditions by producing functional metabolites [1,2]. The literature has reported that each plant accommodates at least one endophyte [3]. Among the endophytes, endophytic fungi have attracted substantial attention as a potential source of biologically active metabolites. They produce metabolites with various biological activities, such as antioxidant, antimicrobial, antiviral, antidiabetic, anticancer, insecticidal, and immunosuppressive. These natural bioactive metabolites are useful for medicinal and pharmaceutical applications [4,5].

Research in endophytic fungi associated with medicinal plants has emerged as an exciting field in discovering novel bioactive metabolites. The literature has shown that medicinal plant–associated endophytic fungi possess structurally diverse chemical entities, such as alkaloids, phenolics, flavonoids, terpenoids, steroids, peptides, and polyketides [6]. In a study conducted by Kusari and colleagues, endophytic fungi– derived metabolites have been reported to possess novel and rare chemical structures. This interesting group of microorganisms is worth extensive investigation in revealing the metabolic interactions of the fungal endophyte with its host to trigger the biosynthesis of bioactive metabolites [7]. Moreover, it has been reported that the chemical profile of medicinal plants could affect the bioactive metabolites derived from fungal endophytes [8]. For instance, Taxol was first isolated as a potent anticancer drug from the bark of the Pacific yew tree (*Taxus brevifolia*). This natural product was then discovered from the yew-associated fungus *Taxomyces andreanae* [9].

*Orthosiphon stamineus* is a perennial herb belonging to the Lamiaceae family. The plant has long been used as a traditional medicine in Southeast Asia for diabetes, hypertension, fever, and jaundice [10]. The literature has reported that *O. stamineus* possesses a high content of phenolics, particularly rosmarinic acid. The presence of rosmarinic acid as the primary phenolic compound in *O. stamineus* has contributed to its excellent antioxidant activity [11,12]. *Diaporthe fraxini* ED2 is an endophytic fungus residing in *O. stamineus*. It was reported that the fungal culture medium supplemented with rosmarinic acid–rich extract stimulated the production of a potent anti-candidal metabolite, phomopsidione [13]. Thus, changes in the culture media and/or conditions could be utilised as an alternative approach to optimise fungal biosynthetic pathways, leading to the production of novel bioactive metabolites [14].

Metabolomics is an emerging analytical technique used to measure and compare the chemical fingerprints of biological samples at a specific condition. It is an essential component of systems biology that focuses on studying metabolites of various metabolic pathways and/or biochemical reactions [15]. In this aspect, the mass spectrometry (MS)-based metabolomics approach offers a unique opportunity for researchers to separate and analyse a vast number of structurally diverse metabolites with important biological information. Liquid chromatography-high resolution mass spectrometry (LC-HRMS)-based metabolomics is favored due to its high throughput, sensitivity, and selectivity. LC-HRMS can detect samples up to nanomolar (nM) concentrations, making it the preferred option for a large scale biological sample study with comprehensive coverage of metabolites [5,16]. Owing to the generation of multiple metabolomics datasets, a statistical approach such as MVDA is employed to aid the understanding of the data trends through visualisation plots [17]. Thus, the present study focused on the discrimination of the chemical profile of *D. fraxini* cultivated in different media through the MS-based metabolomics approach and its antioxidant potential.

## 2. Materials and Methods

### 2.1. Fungal Strain

The endophytic fungus *D. fraxini* ED2 was previously isolated from *O. stamineus* Benth. by Yenn et al., 2017 [13]. It was deposited at Universiti Kuala Lumpur (UniKL), Malaysian Institute of Chemical and Bioengineering Technology (MICET), Melaka, Malaysia. The fungal isolate was cultivated on potato dextrose agar (PDA) and stored at 4 °C prior to use. It was sub-cultured on fresh medium every four weeks to ensure its purity and viability.

### 2.2. Fermentation and Extraction

*D. fraxini* was cultured in two different media: yeast extract sucrose broth (DFC) and yeast extract sucrose broth supplemented with 5 mg/L rosmarinic acid (DFS). The inoculum from each culture medium was prepared by introducing two mycelial agar plugs from seven-day-old fungal culture into an Erlenmeyer flask containing 100 mL of medium. The culture was grown at 30 °C in a shaker at 120 rpm. The fermentative broth and fungal biomass were then separated by centrifugation after 20 days of incubation. Then the supernatant was extracted three times with an equal volume of ethyl acetate (1:1). The organic phase was collected and concentrated to dryness using a rotary evaporator under reduced pressure to yield the fungal extract. The concentrated extract was stored in an amber bottle in a freezer at −80 °C and lyophilized in a freeze dryer. The obtained extract was then stored in an amber bottle in a refrigerator at −20 °C until the analysis. Six individual culture media from each medium group were used as biological replicates to prepare the fungal extracts.

### 2.3. Total Phenolic Content (TPC)

TPC was performed according to the Folin–Ciocalteu method as described by Bobo-Garcia et al. (2015) [18]. In a flat-bottom 96-well microplate, 20 µL of 1 mg/mL extract was mixed with 100 µL of 1:4 diluted Folin–Ciocalteu reagent (R&M Chemicals, Subang, Malaysia) and gently shaken. After 2 min, 75 µL of 100 mg/mL Na_2_CO_3_ (Bendosen, Shah Alam, Malaysia) solution was added and the mixture was briefly shaken. After 2 h of incubation at room temperature, the absorbance was measured at 750 nm. The calibration curve was constructed using gallic acid (Sigma-Aldrich, Burlington, MA, USA) at concentrations ranging from 0.2 to 400 µg/mL. TPC was estimated as gallic acid equivalent (GAE) in µg per mg of extract.

### 2.4. Total Flavonoid Content (TFC)

TFC was measured following Horszwald & Andlauer (2011) with slight modifications [19]. The AlCl_3_ solution was prepared by mixing 10% (*w*/*v*) AlCl_3_.6H_2_O (Fisher Scientific, Waltham, MA, USA), 1 M sodium acetate (Sigma-Aldrich, Burlington, MA, USA), and deionised water in a 1:1:28 (*v*/*v*/*v*) ratio. A total of 100 µL of 1 mg/mL extract prepared in 95% ethanol were mixed with 150 µL freshly prepared AlCl_3_ solution in a 96-well plate and incubated at room temperature for 30 min. The absorbance was then measured at 415 nm against the AlCl_3_ solution as a blank. The standard curve was generated using quercetin (Sigma-Aldrich, Burlington, MA, USA) at concentrations ranging from 1 to 63 µg/mL in 95% ethanol. The results obtained were expressed as µg quercetin equivalent (QE) per mg of extract.

### 2.5. DPPH Free-Radical Scavenging Activity Assay

The DPPH radical scavenging activity was measured in a solvent system buffered at pH 6.0 using a 50 mmol/L sodium phosphate buffer (R&M Chemicals, Subang, Malaysia) and ethanol (95%) at a 1:1 (*v*/*v*) ratio [20]. In a 96-well microplate, 260 µL of DPPH radical (Alfa Aesar, Haverhill, MA, USA) solution (100 µM) was mixed with 40 µL of extract at various concentrations (1–1000 g/mL). The positive control was 0.06–31 µg/mL ascorbic acid (R&M Chemicals, Subang, Malaysia). After 30 min reaction at room temperature, absorbance was taken at 525 nm against the blank (300 µL of solvent). The percentage of DPPH scavenging activity was calculated using the following formula:

$$\mathrm{DPPH}\;\mathrm{scavenging}\;\mathrm{activity}\;\left(\%\right)=\frac{{\mathrm{abs}}_{0}-{\mathrm{abs}}_{\mathrm{extract}}}{{\mathrm{abs}}_{0}}$$

where

abs_0_ = absorbance of negative control
abs_extract_ = absorbance of extract

A plot of % DPPH scavenging activity against concentration was constructed to evaluate the activity–concentration curve. An online tool, QuestGraph^TM^ IC_50_ Calculator, AAT Bioquest, Sunnyvale, CA, USA (https://www.aatbio.com/tools/ic50-calculator, accessed on 24 April 2022) was used to compute the concentration of extract required to reduce the DPPH radical by 50% (IC_50_). The ascorbic acid equivalent antioxidant activity (AAEA) [21] was determined using the IC_50_ value (µg/mL) acquired using the QuestGraphTM IC_50_ Calculator:

$$\mathrm{AAEA}\;\left(µg/\mathrm{mg}\;\mathrm{extract}\right)=\frac{{\mathrm{IC}}_{50}\;\mathrm{AA}}{{\mathrm{IC}}_{50}\;E}\times 10^{3}$$

where

IC_50_ AA = IC_50_ value of ascorbic acid
IC_50_ E = IC_50_ value of extract

### 2.6. Ferric Reducing Antioxidant Power (FRAP) Assay

FRAP assay was performed according to Santos et al. (2017) [20]. The FRAP reagent was made fresh by mixing 300 mM sodium acetate buffer, pH 3.6 (Sigma-Aldrich, Burlington, MA, USA) into 10 mM 2,4,6-tri(2-pyridyl)-1,3,5-triazine (TPTZ) (Acros Organics, Geel, Belgium) prepared in 40 mM HCl (QRec, Rawang, Malaysia) followed by 20 mM FeCl_3_.6H_2_O (Sigma-Aldrich, Burlington, MA, USA) in the ratio of 10:1:1 (*v*/*v*/*v*). In a 96-well microplate, an aliquot of 280 µL FRAP reagent was mixed with 20 µL of 1 mg/mL extract. Absorbance at 593 nm was measured after 30 min of reaction. A standard curve with concentrations ranging 3–200 µg/mL ascorbic acid (R&M Chemicals, Subang, Malaysia) was plotted to evaluate the extract ferric reducing antioxidant power. The results were expressed in µg ascorbic acid equivalent per mg (µg AAE/mg) extract.

### 2.7. ABTS Cation-Radical Reduction Activity Assay

The ABTS cation-radical reduction activity of the samples was determined using the method described by Seo et al. (2015) [22]. The ABTS powder (Roche Life Science, Indianapolis, IN, USA) was first dissolved in deionised water to produce a 7 mM ABTS solution. The ABTS cation-radical was generated during 16 h reaction period with 2.45 mM potassium persulfate (Sigma-Aldrich, Burlington, MA, USA) in the dark at room temperature. Before the assay, the solution was diluted with deionised water to an absorbance of 0.7 at 734 nm. The ABTS cation-radical solution (100 µL) was then added to a 96-well plate containing the 100 µL of 0.005–5000 µg/mL test sample. Trolox (Acros Organics, Geel, Belgium) was used as a positive control, with a concentration of 0.06–31 µg/mL. After 5 min incubation, the absorbance was immediately measured at 734 nm using a microplate reader. The following equation was used to calculate the extract scavenging activity:

$$\mathrm{ABTS}\;\mathrm{scavenging}\;\mathrm{activity}\;\left(\%\right)=\frac{{\mathrm{abs}}_{0}-{\mathrm{abs}}_{\mathrm{extract}}}{{\mathrm{abs}}_{0}}\times 100$$

where

abs_0_ = absorbance of negative control
abs_extract_ = absorbance of extract

The activity–concentration curve was evaluated using a plot of percentage ABTS scavenging activity against concentration. The concentration of extract required for 50% reduction in ABTS radical (IC_50_) was calculated using the QuestGraph^TM^ IC_50_ Calculator. The Trolox equivalent antioxidant activity (TEAA) was calculated using IC_50_ value (µg/mL) obtained from the QuestGraph^TM^ IC_50_ Calculator as follows [21]:

$$\mathrm{TEAA}\;\left(µg/\mathrm{mg}\;\mathrm{extract}\right)=\frac{{\mathrm{IC}}_{50}\;T}{{\mathrm{IC}}_{50}\;E}\times 10^{3}$$

where

IC_50_ T = IC_50_ value of Trolox
IC_50_ E = IC_50_ value of extract

### 2.8. Statistical Analysis

The results were expressed as the means ± standard deviation (SD) of six independent experiments (*n* = 6). The statistical significance of differences between means was established by an unpaired *t*-test using GraphPad Prism 9. *p* values < 0.05 were considered to indicate statistical significance.

### 2.9. LC-HRMS Metabolomic Analysis

Six biological fungal extracts from each DFC and DFS were analysed using an Agilent 1290 Infinity LC system coupled to Agilent 6520 Accurate-Mass Q-TOF mass spectrometer with a dual ESI source. One mg/mL of extract was analysed using an Agilent Zorbax Eclipse column (XDB-C18, Narrow-Bore 2.1 × 150 mm, 3.5 µm) in positive mode. The gradient elution was conducted at the 0.5 mL/min flow rate using purified water (A) and acetonitrile (B) with 0.1% formic acid in each mobile phase. The gradient program started with 5% B and increased gradually to 100% B. The total analysis period for each extract was 25 min. The injection volume was 1 μL, and the column temperature was maintained at 20 °C. HRMS analysis was performed in positive ESI ionisation modes coupled with a spray voltage at 4.0 kV; nitrogen gas was used as the drying gas at 320 °C with a flow rate of 10 L/min, nebuliser pressure: 45 psig, fragmentor voltage: 125 V, and mass range from 100 to 3200 *m*/*z* at a resolving power up to 20,000 (1 s acquisition). The obtained raw MS data files were converted to mzML format using ProteoWizard software (Palo Alto, CA, USA) and MS-DIAL version 4.8 for peak discrimination, filtering, and alignment. Dereplication and metabolite identification for the positive ionisation mode dataset were carried out using the METLIN and DNP databases. The level of identification was L2—putatively identified metabolites through library matching [23]. ChemDraw Professional 20.0 (PerkinElmer, Waltham, MA, USA) software was used for chemical structure drawing.

### 2.10. Multivariate Data Analysis (MVDA)

MetaboAnalyst 5.0 is employed to perform MVDA in the present study. It is a web-based metabolomics data processing platform for statistical, functional, and meta-analyses [24]. A data file (.csv) containing a table with the information of sample name, sample group, peak list, and peak intensity was uploaded onto MetaboAnalyst 5.0 server (https://www.metaboanalyst.ca/, accessed on 24 April 2022). Data were subjected to log transformation and Pareto scaling. Subsequently, univariate analysis was performed followed by multivariate analysis (principal component analysis (PCA), supervised partial least squares-discriminant analysis (PLS-DA), hierarchical clustering, and K-means partitional clustering) [25].

## 3. Results and Discussion

### 3.1. Effects of Culture Medium Supplementation on TPC and TFC

**Figure 1:**
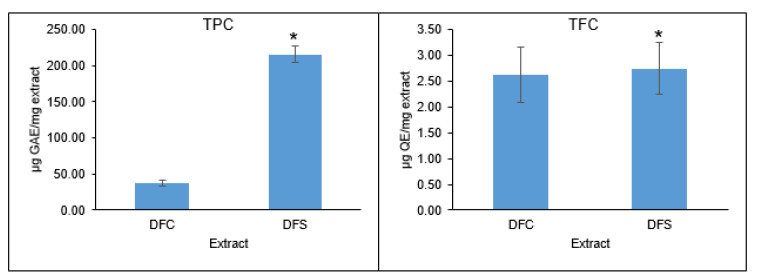
Effects of culture medium supplementation on TPC and TFC in DFC and DFS. Values given are means ± SD, with *n* = 6. * Significantly different from DFC.

The effects of supplementation in the culture medium of *D. fraxini* on the TPC and TFC are shown in Figure 1. Overall, DFS exhibited higher TPC (215.63 µg GAE/mg extract) and TFC (2.74 µg QE/mg extract) than DFC (TPC: 36.98 µg GAE/mg extract; TFC: 2.62 µg QE/mg extract). It is known that phenolic and its derivatives are the major contributors to antioxidant activity by exhibiting free radical inhibition in biological systems. They are regarded as good electron donors due to the presence of aromatic hydroxyl groups that play a key role in scavenging free radicals [26]. According to a study conducted by Verma et al. (2022), *Diaporthe* sp. SAUCC194 extract has been shown to give a TPC of 78.91 µg GAE/mg extract. Thus, different endophytic fungal extracts exhibited different phenolic profiles that could affect their biological activities, particularly antioxidants [27]. According to the literature, flavonoids constituted a small portion of the TPC [28]. The obtained TFC was lower than other fungal extracts from *Diaporthe* sp. [29]. It was reported that fungal endophytes produced bioactive secondary metabolites to inhibit pathogen attacks and self-survival in their specific niches [7]. Thus, a rosmarinic acid–supplemented culture medium may have stimulated the production of related phenolic metabolites in *D. fraxini*, contributing to its high TPC.

### 3.2. Effects of Culture Medium Supplementation on DPPH and ABTS Radical Scavenging and FRAP Reducing Activities

In assessing the antioxidant potential of DFC and DFS, different antioxidant assays were employed. This is due to the fact that complex chemical interactions may occur within the sample. Thus, several assays are warranted to assess different modes of action of antioxidants within a biological system [30]. DPPH, FRAP, and ABTS assays are widely used to evaluate the antioxidant capacities of samples owing to their fast and reproducible results [31]. Generally, DFS showed higher antioxidant activity than DFC in all the assays (Table 1). DPPH assay measures the reducing or scavenging ability of the sample towards the free radicals using the spectrophotometric method. The colour change occurs when there is a reduction of an oxidant. Additionally, the degree of colour change is positively correlated to the concentration of antioxidants in the sample [32]. Based on the results, DFC and DFS recorded an activity of 9.71 ± 2.64 and 332.20 ± 51.07 μg AAE/mg extract, respectively. Additionally, IC_50_ values were obtained and compared with the standard, ascorbic acid. IC_50_ is the amount of sample required for 50% inhibition of a given biological activity. Thus, a lower IC_50_ value exhibited by the sample indicates higher biological activity [33]. The IC_50_ for DFC and DFS were 250.66 and 7.11 µg/mL, respectively, as compared to the standard (IC_50_: 2.31 µg/mL). In a study conducted by Rai et al. (2022), extracts from *Diaporthe tulliensis* and *Diaporthe tectonendophytica* showed an IC_50_ of >200 µg/mL when evaluated using a DPPH assay [34]. Weak activity (IC_50_: >200 µg/mL) was also recorded by fungal extract of *Diaporthe* sp. SAUCC194 isolated from a medicinal plant, *Oroxylum indicum* (L.) Kurz [26]. The present findings highlight the significant DPPH radical scavenging activity displayed by DFS when cultured in a supplemented medium. In FRAP assay, DFS recorded 188.41 ± 18.67 μg AAE/mg extract as compared to DFC (53.88 ± 4.31 μg AAE/mg extract). This assay is based on the reduction of Fe^3+^–TPTZ to give Fe^2+^–TPTZ complex by antioxidants that give an intense blue colour [32]. Meanwhile, the ABTS assay is one of the widely used antioxidant assays. It is also known as the TEAA assay. The stable radical cation ABTS will lose its blue-green colour when reacting with hydrogen-donating antioxidants. It absorbs at a wavelength of 734 nm, which has advantages over the elimination of colour interference and reduction in sample turbidity [35,36]. It is worth noting that DFS (1159.44 ± 67.70 μg TE/mg extract) showed an exceptionally higher antioxidant potential than DFC (37.77 ± 6.13 μg TE/mg extract). In addition, DFS (IC_50_: 17.83 μg/mL) exhibited potent IC_50_ as compared to DFC (IC_50_: 560.32 μg/mL) and the standard trolox (IC_50_: 20.61 μg/mL) in ABTS assay. Compared to fungal extracts from *Diaporthe* sp. isolated from mangrove plants which displayed IC_50_ ranging from 0.77 to 13.56 mg/mL, the present findings suggested the potential of DFS as a source of natural antioxidants [37].

### 3.3. LC-HRMS-Based Metabolomics Analysis

**Figure 2:**
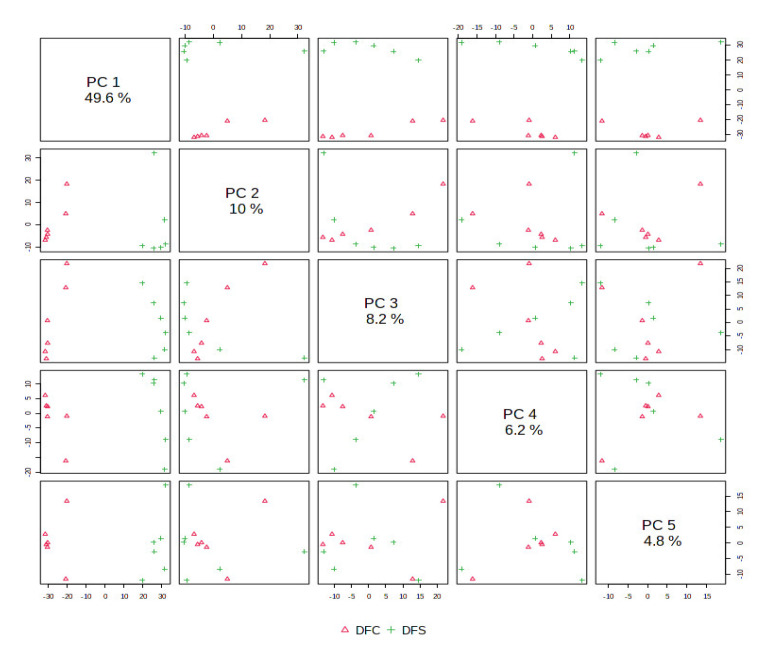
PCA pairwise score plot in unsupervised analysis of DFC and DFS. Different PCs illustrate variability in the spatial distribution of the sample groups.

**Figure 3:**
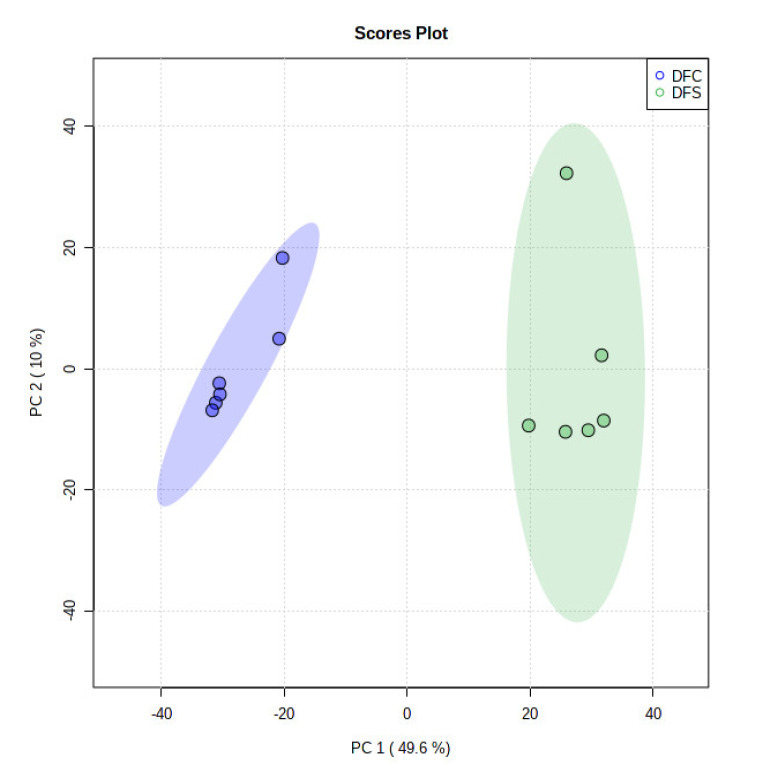
PCA scores plot in unsupervised analysis. PC1 versus PC2 showing the discrimination of DFC and DFS growing in different culture media.

**Figure 4:**
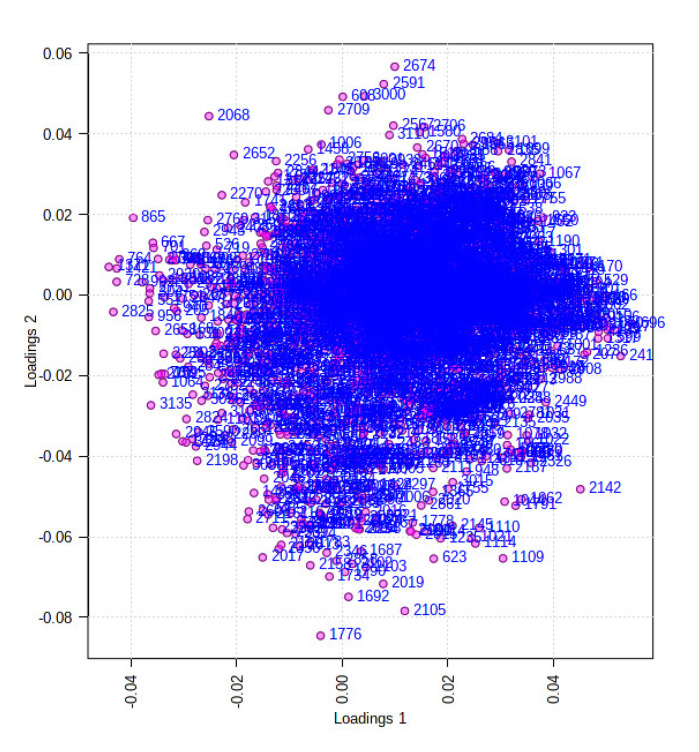
PCA loading plot in unsupervised analysis obtained from DFC and DFS. Shown data are the Var ID (peak number).

**Figure 5:**
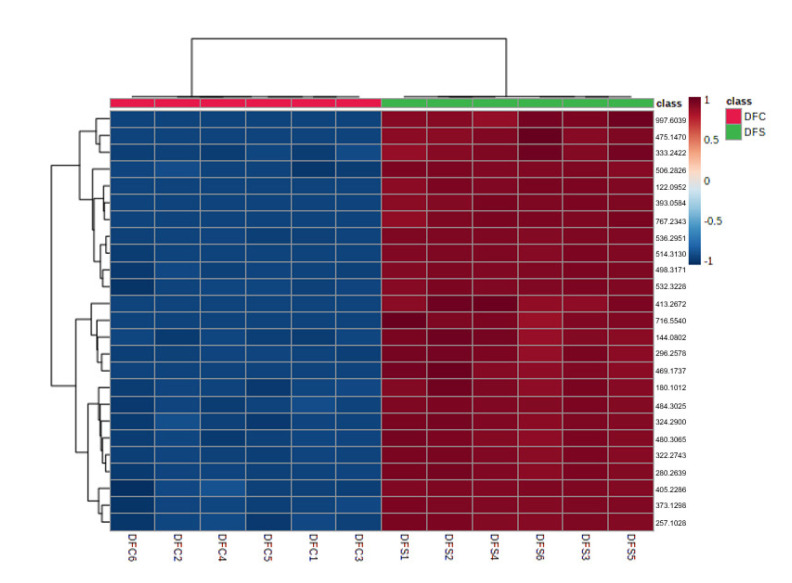
Heatmap overview showing the discriminations of DFC and DFS. The colour scale was set to default ranging from red (high) to blue (low).

**Figure 6:**
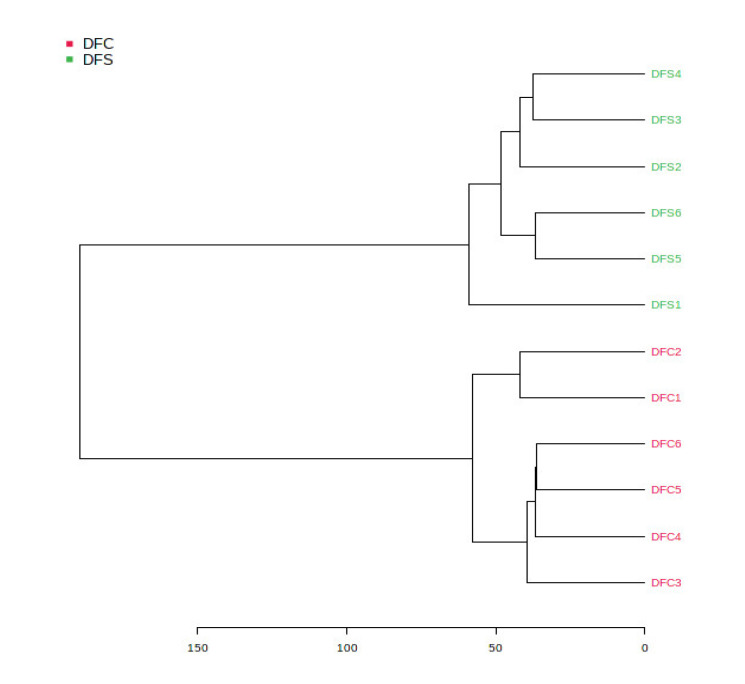
HCA plot showed as dendrogram in unsupervised analysis for DFC and DFS corresponding to the PCA model.

The ethyl acetate extracts of *D. fraxini* grown in different media were analysed in positive ion mode using LC-HRMS. An untargeted metabolomics approach was conducted to characterize the metabolites present in the 12 extracts by considering the low molecular weight ionisable molecules. Six independent biological replicates were used for each culture medium to explore the metabolome differences between the fungal extracts. A total of 3164 features were detected in the 12 fungal extracts. The dataset from LC-HRMS analysis was subjected to MetaboAnalyst 5.0 to interpret and analyse the large metabolomics data generated from the samples. In unsupervised analysis, principal component analysis (PCA) was performed to distinguish the key differences between the sample groups. Generally, it is used to reduce many dimensionalities into important essential factors, thus providing an overview of the dataset [38]. Figure 2 shows the PCA pairwise score plots between the principal components (PCs). It was depicted that five PCs explained 78.8% of the total variation. In detail, PC1 and PC2 contributed to 59.6% of the total variation while PC1, PC2, and PC3 accounted for 67.8% of the total variation. Different culture media extracts were clearly distinguished based on the PCA 2D scores plot (Figure 3). The fungal extracts DFC and DFS were distributed into two distinct areas indicating these two extracts are statistically different from each other. It was observed that the six replicates from each DFC and DFS showed the scores closely, indicating the reproducibility of the fungal culture extracts. Moreover, the PCA was depicted in loading plot to distinguish the discrimination of the metabolites (Figure 4). The PCA analysis exhibited clear discrimination of the fungal extracts DFC and DFS, suggesting their unique patterns as shown in the heatmap (Figure 5). In clustering analysis, it was distinctive that samples were grouped into two main clusters. Hierarchical cluster analysis (HCA) demonstrated a better distance matrix of the relationship between the culture extracts. HCA was found in accordance with the PCA results and allowed a better resolution of classification of the sample replicates [39]. HCA utilises a similarity metric between pairs of samples to give a dendrogram of nested clusters [40]. Based on the dendrogram (Figure 6), the vertical axis showed the arrangement of the cluster while the horizontal axis showed the clusters’ similarity. In this aspect, each sample begins with its own cluster and similar clusters will then be merged as the hierarchy shifts to the left [41]. Thus, HCA further strengthened the PCA findings that DFC and DFS were well discriminated. According to previous literature, the metabolite variations were noticed among the different fungal cultures. Fungal interactions were believed to be the determining factor in contributing to the differences of the metabolite profiles [42]. In a study conducted by Tawfike and co-workers, different culture extracts of endophytic *Curvularia* sp. showed changes in their chemical profiles when examined using heatmap analysis. On different culture media, the occurrence of the metabolites was well discriminated, indicating chemical diversity of the studied fungal extracts [43].

**Figure 7:**
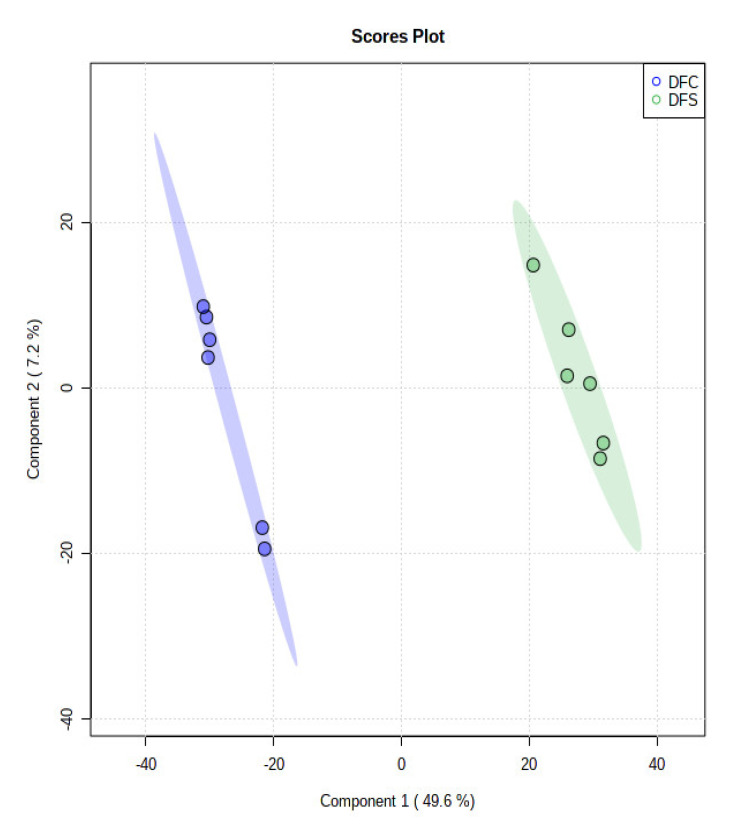
PLS-DA scores plot in supervised analysis. Predictive components 1 versus 2 showing the supervised separation between the two sample groups (DFC and DFS) based upon the culture supplementation.

**Figure 8:**
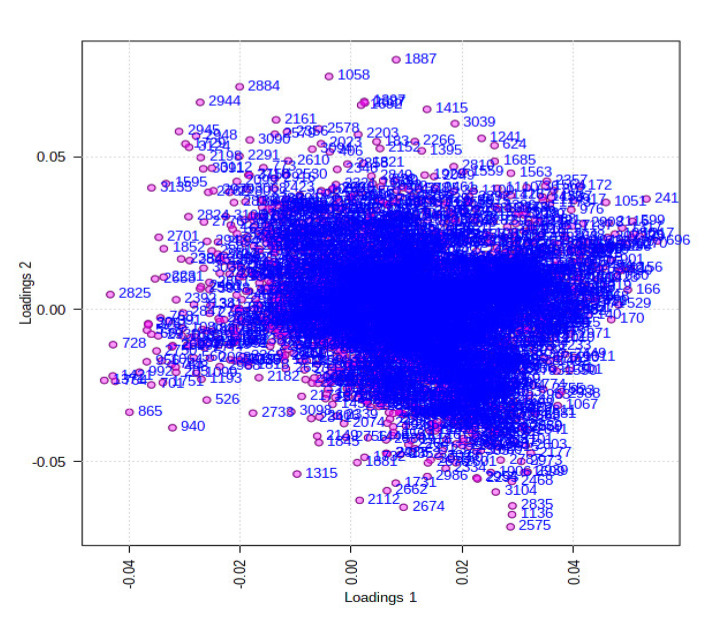
PLS-DA loading plot in supervised analysis obtained from DFC and DFS. Shown data are the Var ID (peak number).

**Figure 9:**
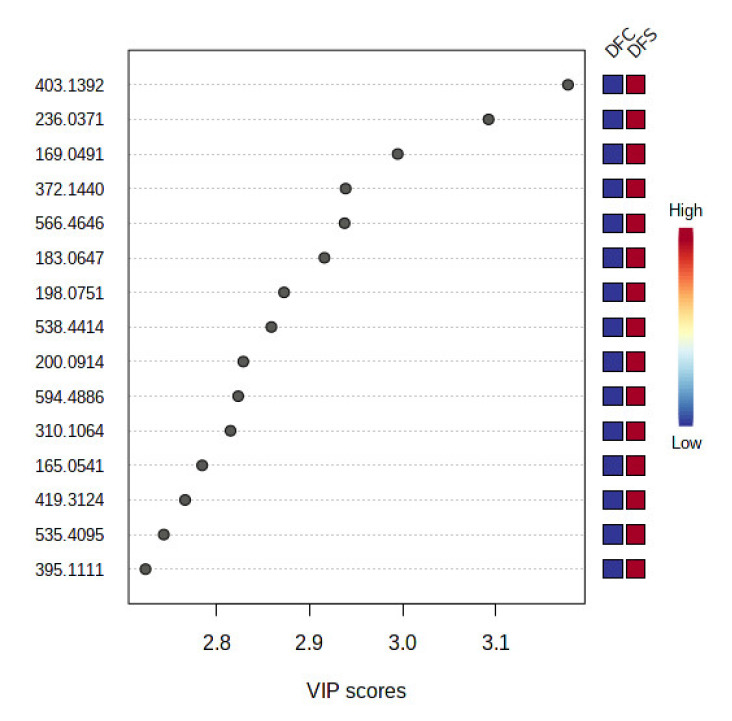
VIP score plot of the most important discriminant metabolites by PLS-DA for DFC and DFS. The relative abundance of each important metabolite is indicated with a colour code scaled from blue (low) to red (high). A high VIP score indicates a high impact of the metabolite as a discriminant feature among the sample groups.

**Figure 10:**
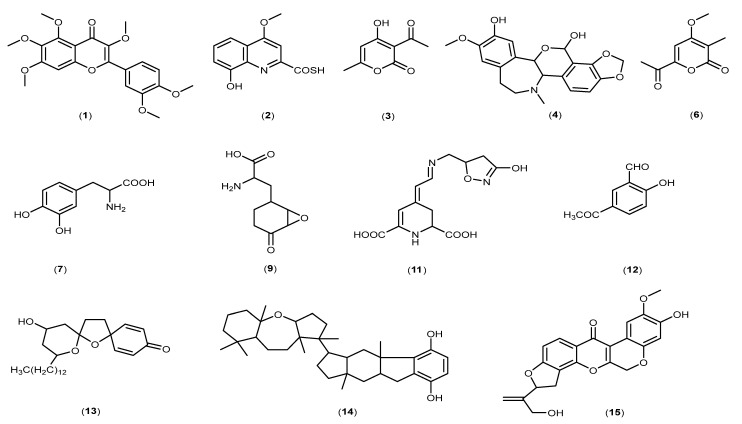
Chemical structures of discriminant putatively identified metabolites in DFC and DFS.

In supervised analysis, partial least squares-discriminant analysis (PLS-DA) is performed to distinguish biological samples within-group variation from between-group variation. It is a widely used supervised technique that integrates the extracted features and discriminants into one algorithm [44]. The accuracy, correlation coefficient R2 as well as the cross-validation correlation coefficient Q2 of the dataset were more than 0.8, suggesting good predictability of the model. PLS-DA 2D scores and loading plots are shown in Figure 7 and Figure 8. In PLS-DA scores plot, 56.8% of the total variations were explained by the two PLS components. The first and second components recorded 49.6% and 7.2%, respectively. In addition to that, PLS-DA established the 15 highest values of variable importance in projection (VIP) scores, as shown in Figure 9. The most 15 important features were shown on the vertical axis in an ascending order. These metabolites were dereplicated using METLIN and DNP to give 12 metabolites (Figure 10) and three unknowns (Table 2). The [M+H]^+^ ion peaks at *m*/*z* 403.1392, 236.0371, 169.0491 and 372.1440 suggested the presence of hexamethylquercetagetin (C_21_H_22_O_8_) (**1**), thioquinolactobactin (C_11_H_9_NO_3_S) (**2**), 3-acetyl-4-hydroxy-6-methyl-2H-pyran-2-one (C_8_H_8_O_4_) (**3**) and *N*-methyl-14-*O*-demethylepiporphyroxine (C_20_H_21_NO_6_) (**4**), respectively. Hexamethylquercetagetin was previously isolated as a new metabolite in *Citrus* plant [45], while thioquinolactobactin was found as a siderophore from *Pseudomonas*. This metabolite possessed significant antimicrobial activity with iron-chelating property [46]. 3-Acetyl-4-hydroxy-6-methyl-2H-pyran-2-one, also known as methylacetopyronone, was detected in the chloroform extract of *Solandra nitida* [47]. Meanwhile, *N*-methyl-14-*O*-demethylepiporphyroxine is an alkaloid previously found in *Papaver somniferum*. This medicinal plant contains various alkaloids with potent pharmacological and bioactive properties [48]. Vermopyrone (**6**), an α-pyrone, was first isolated from the fungus *Gliocladium vermoesenii* [49]. This metabolite was also present in *Cephalotaxus hainanensis* for anticancer activity [50]. Metabolite 2-amino-3-(3,4-dihydroxyphenyl)propanoic acid (**7**) is a catechol α-amino acid. Commonly, it is known as 3,4-dihydroxyphenylalanine and is used to treat Parkinson’s disease [51]. A [M+H]^+^ peak at *m*/*z* 200.0914 suggested the presence of α-amino-5-oxo-7-oxabicyclo[4.1.0]heptane-2-propanoic acid (anticapsin) (**9**). This metabolite was found to act as an inhibitor of glucosamine synthetase in *Staphylococcus aureus* [52]. Additionally, 12-decarboxy-4′,5′-dihydromuscaaurin I (**11**) was reported as a pigment from *Amanita muscaria* [53], while 5-acetyl-2-hydroxybenzaldehyde (**12**) was found as a new *p*-hydroxyacetophenone derivative from *Senecro graveolens* [54]. Metabolite aculeatin A (**13**) was isolated as a novel dioxadispiro[5.1.5.2]pentadeca-9,12-dien-11-one derivative from *Amomum aculeatum*. It showed a potent cytotoxic effect against KB cells and anti-protozoal activities on *Plasmodium* strains [55]. Interestingly, metabolite toxicol B (**14**) was previously isolated as a novel structure from the extract of *Toxiclona toxius*, which showed activity on human immunodeficiency virus type 1 (HIV-1) reverse transcriptase (RT) [56]. 3-*O*-Demethyldehydroamorphigenin (**15**) was previously isolated as a bioactive phenolic from the fruits of *Amorpha fruticosa*. Nonetheless, the metabolite exhibited weak antibacterial and cytotoxic activities [57]. The literature has reported that the fungal metabolomes depend on various experimental parameters. To date, there are various techniques to conduct metabolomics analyses to identify the fungal metabolites from the extracts [58]. Plant-associated fungal endophytes could produce bioactive metabolites that may be used as therapeutic agents against several diseases. The possibilities of discovering novel bioactive metabolites are exciting, particularly in unknown structures. Thus, searching for novel chemical skeletons from endophytic fungi is essential for the sustainable production of desirable natural products [7].

## 4. Conclusions

Plant-associated fungal research has developed over the past decades along with the advancement of new technologies. In this aspect, metabolomics is tremendously contributing to the generation of comprehensive information to uncover the metabolomes of a fungal biological system. It is an indispensable technique used to study the global fungal metabolites at a particular condition and/or state. Moreover, MVDA in a metabolomics study is crucial to modeling variances, and thus, to presenting an overview of the dataset. An untargeted metabolomics approach was performed by employing an LC-HRMS to characterize the fungal extracts of *D. fraxini* grown in different culture media. A supplemented culture medium of *D. fraxini* exhibited potent antioxidant activity when evaluated using DPPH, ABTS, and FRAP assays. Metabolites discrimination was generated using unsupervised analysis for visual representation and explorative study. Additionally, the application of supervised PLS-DA analysis allowed the extraction of important metabolites features, which contributed to the discrimination of the fungal culture media. These metabolite markers are warranted for targeted metabolomics profiling for specific culture media conditions. The present study offers an important reference to producing bioactive metabolites from fungal endophytes residing in medicinal plants.

## Figures and Tables

**Table 1:** Effects of culture medium supplementation on DPPH and ABTS radical scavenging and FRAP reducing activities in DFC and DFS.

Extract	DPPH (μg AAE/mg Extract)	FRAP (μg AAE/mg Extract)	ABTS (μg TE/mg Extract)
DFC	9.71 ± 2.64	53.88 ± 4.31	37.77 ± 6.13
DFS	332.20 ± 51.07 *	188.41 ± 18.67 *	1159.44 ± 67.70 *

Values given are means ± SD, with *n* = 6. * Significantly different from DFC.

**Table 2:** Dereplication of discriminant putatively identified metabolites in DFC and DFS. The level of identification was L2—putatively identified metabolites through library matching [23].

No.	Retention Time	[M+H]^+^	Molecular Mass		Mass Error (mDa)	Molecular Formula	Putative Identification
			Observed	Calculated			
**1.**	12.61	403.1392	402.1319	402.1315	0.4	C_21_H_22_O_8_	Hexamethylquercetagetin
**2**.	12.24	236.0371	235.0298	235.0303	−0.5	C_11_H_9_NO_3_S	Thioquinolactobactin
**3**.	7.27	169.0491	168.0418	168.0423	−0.5	C_8_H_8_O_4_	3-Acetyl-4-hydroxy-6-methyl-2H-pyran-2-one
**4**.	9.81	372.1440	371.1367	371.1369	−0.2	C_20_H_21_NO_6_	*N*-Methyl-14-*O*-demethylepiporphyroxine
**5**.	22.16	566.4646	-	-	-	-	Unknown
**6**.	7.00	183.0647	182.0574	182.0579	−0.5	C_9_H_10_O_4_	Vermopyrone
**7**.	7.00	198.0751	197.0678	197.0688	−1.0	C_9_H_11_NO_4_	2-Amino-3-(3,4-dihydroxyphenyl)propanoic acid
**8**.	20.71	538.4414	-	-	-	-	Unknown
**9**.	7.00	200.0914	199.0842	199.0845	-0.3	C_9_H_13_NO_4_	α-Amino-5-oxo-7-oxabicyclo[4.1.0]heptane-2-propanoic acid
**10**.	17.24	594.4886	-	-	-	-	Unknown
**11**.	10.76	310.1064	309.0991	309.0961	3.0	C_13_H_15_N_3_O_6_	12-Decarboxy-4′,5′-dihydromuscaaurin I
**12**.	7.00	165.0541	164.0468	164.0473	−0.5	C_9_H_8_O_3_	5-Acetyl-2-hydroxybenzaldehyde
**13**.	17.24	419.3124	418.3051	418.3083	−3.2	C_26_H_42_O_4_	Aculeatin A
**14**.	23.46	535.4095	534.4022	534.4073	−5.1	C_36_H_54_O_3_	Toxicol B
**15**.	12.25	395.1111	394.1038	394.1053	−1.5	C_22_H_18_O_7_	3-*O*-Demethyldehydroamorphigenin

## Data Availability

Not applicable.

## Abbreviations

AAEA	Ascorbic acid equivalent antioxidant activity
ABTS	2,2-Azinobis (3-ethylbenzothiazoline-6-sulfonic acid
DFC	*D. fraxini* cultured in yeast extract sucrose broth
DFS	*D. fraxini* cultured in yeast extract sucrose broth supplemented with 5 mg/L rosmarinic acid
DNP	Dictionary of Natural Products
DPPH	2,2-Diphenyl-1-picrylhydrazyl
FRAP	Ferric reducing antioxidant power
GAE	Gallic acid equivalent
HCA	Hierarchical cluster analysis
HIV-1	Human immunodeficiency virus type 1
LC-HRMS	Liquid chromatography-high resolution mass spectrometry
MS	Mass spectrometry
MVDA	Multivariate data analysis
nM	Nanomolar
PCs	Principal components
PCA	Principal component analysis
PDA	Potato dextrose agar
PLS-DA	Partial least squares-discriminant analysis
QE	Quercetin equivalent
RT	Reverse transcriptase
SD	Standard deviation
TE	Trolox equivalent
TEAA	Trolox equivalent antioxidant activity
TFC	Total flavonoid content
TPC	Total phenolic content
TPTZ	2,4,6-Tri(2-pyridyl)-1,3,5-triazine
VIP	Variable importance in projection
